# Biofilm-Formation in Clonally Unrelated Multidrug-Resistant *Acinetobacter baumannii* Isolates

**DOI:** 10.3390/pathogens9080630

**Published:** 2020-08-02

**Authors:** Aisha M. Alamri, Afnan A. Alsultan, Mohammad A. Ansari, Amani M. Alnimr

**Affiliations:** 1 Department of Clinical Laboratory Sciences, College of Applied Medical Sciences, Imam Abdulrahman Bin Faisal University, Dammam 34212, Saudi Arabia; afalsultan@iau.edu.sa; 2 Department of Epidemic Disease Research, Institute of Research and Medical Consultations (IRMC), Imam Abdulrahman Bin Faisal University, Dammam 31441, Saudi Arabia; 3 Department of Microbiology, College of Medicine, Imam Abdulrahman Bin Faisal University, Dammam 34212, Saudi Arabia; amalnimr@iau.edu.sa

**Keywords:** enterobacterial repetitive intergenic consensus-PCR genotyping, risk factors, antimicrobial susceptibility, nosocomial pathogen

## Abstract

This study analyzed the genotype, antibiotic resistance, and biofilm formation of *Acinetobacter baumannii* strains and assessed the correlation between biofilm formation, antibiotic resistance, and biofilm-related risk factors. A total of 207 non-replicate multi-drug-resistant *A. baumannii* strains were prospectively isolated. Phenotypic identification and antimicrobial susceptibility testing were carried out. Isolate biofilm formation ability was evaluated using the tissue culture plate (TCP), Congo red agar, and tube methods. Clonal relatedness between the strains was assessed by enterobacterial repetitive intergenic consensus-PCR genotyping. Of the 207 isolates, 52.5% originated from an intensive care unit setting, and pan resistance was observed against ceftazidime and cefepime, with elevated resistance (99–94%) to piperacillin/tazobactam, imipenem, levofloxacin, and ciprofloxacin. alongside high susceptibility to tigecycline (97.8%). The Tissue culture plate, Tube method, and Congo red agar methods revealed that 53.6%, 20.8%, and 2.7% of the strains were strong biofilm producers, respectively, while a significant correlation was observed between biofilm formation and device-originating respiratory isolates (*p* = 0.0009) and between biofilm formation in colonized vs. true infection isolates (*p* = 0.0001). No correlation was detected between antibiotic resistance and biofilm formation capacity, and the majority of isolates were clonally unrelated. These findings highlight the urgent need for implementing strict infection control measures in clinical settings.

## 1. Introduction

*Acinetobacter baumannii* is an opportunistic nosocomial pathogen, frequently causing various infections in humans, including sepsis, meningitis, peritonitis, urinary tract-, soft-tissue-, and device-related infections, such as ventilator-associated pneumonia [1].

Antimicrobial resistance represents a great challenge in *A. baumannii* isolates and is reported worldwide with notable resistance to major classes and the most frequently utilized antimicrobial agents, including β-lactams, aminoglycosides, and fluoroquinolones [2,3]. Due to the elevated incidence of multidrug-resistant *A. baumannii* (MDRAB), extended-spectrum β-lactams, such as carbapenems, of which meropenem and imipenem are categorized as the ‘most effective’ therapeutic options, are currently used to treat complicated infections. Unfortunately, reports indicating the elevated *A. baumannii* resistance to carbapenems are on the rise, which limits the treatment options to drugs known for their neurotoxicity and nephrotoxicity, such as Colistin [4].

Most of these strains contain carbapenem-hydrolyzing β-lactamase genes (CHDLs), such as class D and class B metallo-β-lactamases (MBLs) [4]. Along with its elevated multidrug resistance, the ability of *A. baumannii* to produce microbial biofilms has caused serious global problems by contributing toward its survival and transmission in hospital environments on biotic and abiotic surfaces, including cerebrospinal fluid shunts and catheters [5].

Microbial biofilms are assemblies of microorganisms within a matrix that functions cooperatively to provide a protected microbial niche and enhanced resistance to various antimicrobial agents by reducing drug diffusion through bacterial cells, thus facilitating the survival of clinical isolates under severe environmental conditions with multidrug resistance [6]. Microcolonies in biofilms are complex bacterial communities with intraspecies communication and adaptation through quorum-sensing, which regulates virulence [7]. *A. baumannii* is reportedly tolerant to extracellular stressors in biofilms in the skin and in soft tissue infections, both within the wound and on occlusive dressings [8]. *A. baumannii* can also form biofilms on most abiotic surfaces, including hospital surfaces and equipment, such as ventilator tubes, catheters, and stainless steel [5]. Strong biofilm-producing *A. baumannii* strains are less sensitive to dehydration than weak biofilm-producing strains; thus, biofilm production is critical for the organism’s survival under dry conditions [6,7].

Although bacteria within biofilm communities express adhesins and surface factors, including capsular polysaccharides, which contribute to biofilm formation and maturation, these components are poorly understood [9]. Factors enhancing biofilm formation appear strain-dependent; however, some have been evaluated, with most biofilm-producing *A. baumannii* strains associated with intensive care unit (ICU) admission and the use of medical devices [8,10]. In this study, we investigated antibiotic susceptibility, biofilm formation, and clonal relatedness of various clinical multidrug-resistant (MDR) *A. baumannii* isolates. Moreover, we compared three different biofilm assessment methods and evaluated the correlation between antibiotic resistance and biofilm formation.

## 2. Results

### 2.1. Isolate Characterization

The clinical and epidemiological characteristics of 207 MDR *A. baumannii* isolates were analyzed. The strains were isolated from patients aged 2–98 years (mean, 51 years), mostly from male patients (*n* = 128, 61.8%). MDR *A. baumannii* isolates were largely clustered in patients aged 45–74 years (*n* = 94, 45.4%), with children aged <15 years (*n* = 7, 3.4%) displaying the least distribution (Table 1). Most MDR *A. baumannii* strains were isolated from respiratory specimens (*n* = 97, 46.9%), followed by skin and soft tissue specimens (*n* = 66, 31.9%). Blood isolates accounted for 16 samples (7.7%), and other strains were isolated from urine and sterile body fluids (*n* = 28, 13.5%). Of the 207 MDR isolates, most (52.5%) were recovered from the ICU, followed by medical units (26.8%) and surgical units (20.8%).

### 2.2. Antibiotics Susceptibility Testing (AST)

All 207 isolates were categorized as MDR, with resistance to ceftazidime and cefepime (*n* = 207, 100%) and to piperacillin/tazobactam and ciprofloxacin (n = 205, 99%). Furthermore, high carbapenem resistance, evident from increased resistance to imipenem (*n* = 203, 98.1%) and meropenem (*n* = 131, 63.3%), was observed. Other AST profiles revealed 94.7% resistance to levofloxacin and similar resistance to gentamycin and amikacin (*n* = 103, 49.8%). Moreover, most isolates displayed trimethoprim resistance (*n* = 187, 90.3%) but showed the lowest resistance to tigecycline (*n* = 17, 8.2%).

### 2.3. Biofilm Formation among Clinical MDR A. baumannii Isolates

Of the 207 clinical MDR *A. baumannii* isolates, 183 were screened for biofilm production using the Tube method (TM), Congo red agar (CRA), and standard Tissue culture plate (TCP) methods. Through the reference TCP method, most of these isolates (98, 53.6%) were categorized as strong biofilm producers, while 72 (39.3%) were categorized as moderate, and 13 (7.1%) lacked biofilm formation abilities. The TM revealed that 38 (20.8%) isolates were strong biofilm producers, 54 (29.5%) were moderate biofilm producers, and 91 (49.7%) were weak/non-biofilm producers. Conversely, the CRA method revealed that five (2.7%) isolates were strong biofilm producers, seven (3.8%) displayed an intermediate biofilm production phenotype, and the remaining 171 (93.4%) were considered non-biofilm producers.

### 2.4. Biofilm Test Performance against the Gold Standard TCP

To compare the performance of each biofilm formation assay with the reference TCP method, we estimated their sensitivity, specificity, positive predictive value (PPV), negative predictive value (NPV), and overall test accuracy (Table 2). Although the CRA method reported satisfactory specificity (92.86%) and PPV (92.37%), it reported poor sensitivity (6.51%) and a low NPV (6.96%). Conversely, the TM reported average sensitivity (52.73%) and specificity (72.22%), elevated PPV (96.21%), and a poor NPV (10.31%). Moreover, the overall accuracy of the TM and CRA method was significantly inferior to that of the gold standard TCP method (50.09 and 12.55%, respectively).

### 2.5. Biofilm Production in Device- and Non-Device-Related Isolates

**Figure 1:**
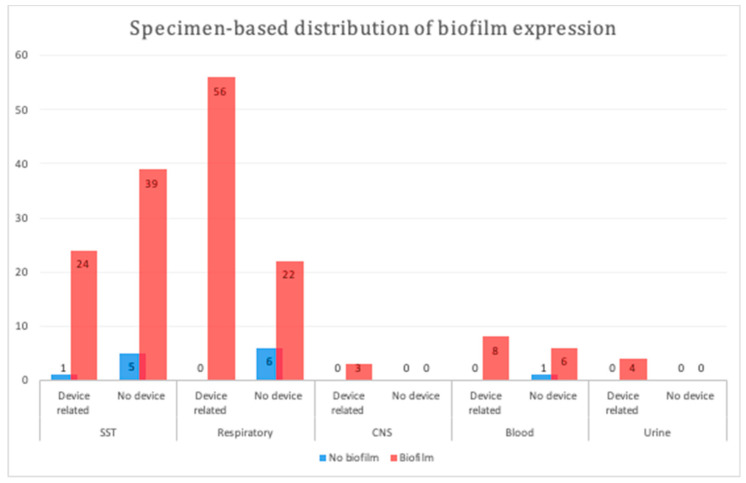
Specimen-based distribution of biofilm production in different samples. A statistically significant difference was observed between device- and non-device-related respiratory samples (*p* = 0.0009). SST, skin and soft tissue; CNS, central nervous system.

We compared the biofilm formation ability of device- and non-device-related MDR *A. baumannii* isolates among the major specimen categories herein. Device-related isolates originating from respiratory samples displayed a significantly higher biofilm formation ability (*p* = 0.0009; Figure 1); however, no significant difference was observed between the device- and non-device-related isolates among the other types of samples (*p* = 1 to *p* = 0.4).

### 2.6. Antimicrobial Resistance and Biofilm Production

**Figure 2:**
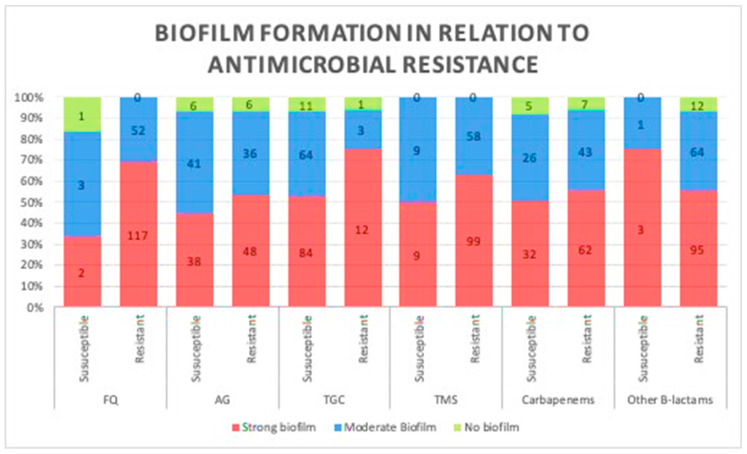
Association between biofilm formation capacity and antibiotic resistance. No relationship was observed between biofilm formation and resistance to different classes of antibiotics. (Data presented is based on the reference Tissue culture plate method FQ, fluoroquinolones; AG, aminoglycosides; TGC, tigecycline; TMS, trimethoprim.

A comparison between biofilm formation, based on the results of the TCP method in both antibiotic-resistant and -susceptible strains, was carried out to investigate the correlation between biofilm formation ability and the level of antibiotic resistance, and no significant difference was observed in their biofilm production potentials (*p* = 0.12; Figure 2). Therefore, we assessed any potential relationship between biofilm formation and the number of antibiotics each strain was susceptible to; similarly, no significant difference was observed, as more susceptible strains produced similar patterns of biofilms to less susceptible strains (*p* = 0.2).

### 2.7. Biofilm Formation Versus Colonization, Infection Status, and Location

**Figure 3:**
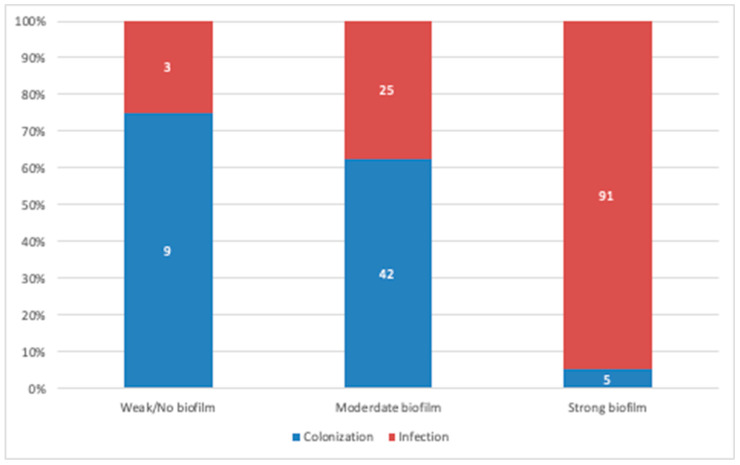
Comparative analysis between biofilm producers originating from colonization and infection cases. A strong positive correlation was observed between biofilm production and strains originating from infection cases. (Data presented is based on the reference TCP method).

On comparing biofilm production levels in isolates originating from colonization and true infections, the strains isolated from true infections displayed a significantly higher biofilm production potential (*p* = 0.0001; Figure 3). Furthermore, we assessed the location of isolates as a potential predictor of biofilm formation; however, no correlation was observed, as biofilm producers were distributed randomly throughout the ICU, medical, and surgical units (*p* = 0.47).

### 2.8. Genotyping of A. baumannii Isolates

**Figure 4:**
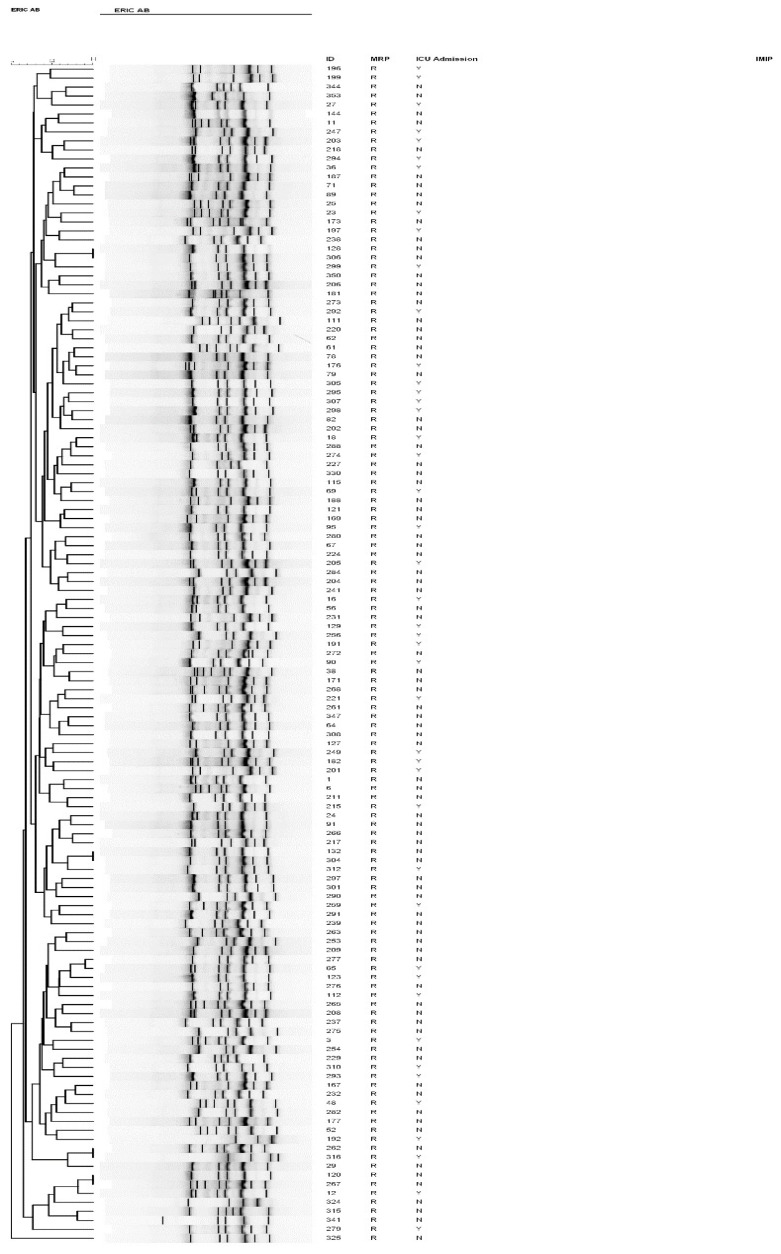
Dendrogram of all carbapenem-resistant *A. baumannii* isolates from patients admitted to intensive care units between 2016 and 2018, as typed by ERIC-PCR. Numbers indicate the corresponding strains, followed by meropenem susceptibility status.

To characterize the genotype of the *A. baumannii* isolates, we performed ERIC-PCR fingerprinting for a subset of the strains, the meropenem-resistant isolates, which grouped them into different clusters (Figure 4). Of the 131 strains genotyped, four pairs of strains shared a similar ERIC-PCR pattern and were found to be genetically related (Table 3).

Similar clones originated from various specimens isolated from different locations with different chronological spans. Despite being clonally related, some clusters displayed slight differences in their AST profiles to the two aminoglycosides, gentamicin, and amikacin, in addition to trimethoprim (Table 3). Interestingly, despite being chronologically apart, identical clusters had comparable biofilm-forming capacity.

## 3. Discussion

MDR *A. baumannii* is an opportunistic pathogen associated with several outbreaks in healthcare and community settings. Herein, we isolated 207 MDR *A. baumannii* strains from an academic medical center over 3 years, of which most were obtained from respiratory specimens, and all were resistant to one or more of ≥3 classes of antibiotics, fulfilling the MDR or extensively drug-resistant (XDR) consensus definition [11].

Tigecycline is one of the few drugs effective against infections due to XDR *A. baumannii*, for which no Clinical laboratory standards institute (CLSI) breakpoints have been reported thus far. In our cohort, some isolates demonstrated high minimum inhibitory concentrations (MICs) against this drug (2 μg/mL); however, the AST methods commonly used as diagnostic tools, such as E-test and automated systems (e.g., VITEK 2), can potentially overestimate tigecycline MICs [12]. Herein, we observed high overall resistance to most of the antimicrobial agents, including extended-spectrum β-lactams, carbapenems, and fluoroquinolones; however, strains demonstrated high susceptibility to tigecycline, which is the “drug of choice” for complex infections caused by MDR Gram-negative bacteria [13]. While tigecycline is mainly used to treat certain infected sites and is not indicated in cases of bactermia [14], studies published in Saudi Arabia and other Gulf Cooperation Council (GCC) countries have reported variable levels of tigecycline resistance among *A. baumannii* strains [15,16]. In the present MDR strains, tigecycline was an effective therapeutic agent with a susceptibility level of >95%, being considerably greater than that estimated in various local studies (20–70%) [2,3]. Although half of the isolates were resistant to aminoglycoside, studies in our region have reported different levels of gentamicin and amikacin resistance (25–96%) [4], while increased aminoglycoside resistance has been reported in studies in China and Egypt [17,18]. Furthermore, very high fluoroquinolone resistance levels were observed in our isolates, concurrent with previous reports from other GCC countries and Turkey [4,19].

Furthermore, in this study, we compared the performance of various methods for assessing biofilm production. Concurrent with previous reports, we found that the performance of the TCP method was superior to that of TM and CRA methods for qualitative analysis of biofilm formation [20,21]. Moreover, these methods revealed that strong and moderate biofilm production was markedly higher in the present cohort (53.6% and 39.3%, respectively), concurrent with previous studies reporting a significant cluster of biofilm-producing *A. baumannii* isolates [22,23]. Furthermore, we investigated the association between the antibiotic resistance phenotype and biofilm formation in the *A. baumannii* clinical isolates by comparing biofilm formation in resistant and susceptible strains; however, no significant difference was observed, probably because the present cohort contained MDR strains that are only susceptible to few antimicrobial agents among those tested herein. Notably, biofilm formation was independently associated with the number of susceptible drugs; therefore, susceptibility to more than one antibiotic did not reduce the biofilm formation efficiency (*p* = 0.2).

Consistently, previous studies have reported an inverse correlation between antibiotic resistance and biofilm formation in *A. baumannii*, wherein biofilm-forming isolates are significantly more susceptible to various antimicrobial agents, including amikacin, gentamicin, ceftazidime, cefepime, ciprofloxacin, imipenem, and meropenem [24,25]. One such report was generated from a multicenter study in 2010–2013 and included 272 *A. baumannii* isolates with variable antibiotic susceptibility [24]. Using a single biofilm detection method (crystal violet, similar to TM in this study), their study revealed that 79.4% of the strong biofilm-producers were non-resistant isolates, whereas 20.6% were MDR/XDR [24]. Another study evaluated the adhesive features of *A. baumannii* isolates from the sputum of 121 cases of hospital-acquired pneumonia, revealing significantly lower biofilm formation among strains with high gentamicin, minocycline, and ceftazidime resistance [26]. Conversely, Yang et al. reported that highly resistant isolates were strongly associated with biofilm formation, with resistance to certain antibiotics, including penicillin, efficiently promoting biofilm production [23], probably owing to the activation of genes involved in biofilm formation, thus providing these resistant strains a fitness advantage [8]. Although both resistant and susceptible isolates included in our study were found to produce biofilms at the same efficiency, a correlation between biofilm-producing capacity and antibiotics resistance was hard to conclude because our cohort comprised of highly resistant isolates ranging from MDR to XDR with variable antimicrobial resistance profile, so further work is needed to assess the biofilm-forming ability between highly resistant isolates relative to fully susceptible strains from a similar geographical area. Together with these previous reports, our results suggest that biofilm-forming strains rely less on antimicrobial resistance to survive and indicate an independent association between antibiotic resistance and biofilm formation.

In our cohort, strains isolated from infection cases presented a significantly higher biofilm production potential than those originating from colonization sites. To our knowledge, this is the first study that compared biofilm formation between colonization and infection isolates in clinical strains of MDR *A. baumannii*. However, isolate’s location was not correlated with biofilm formation ability as those from the ICU did not tend to produce more biofilms than isolates from other hospital settings. These findings are consistent with those of Rodríguez-Baño, who reported that ciprofloxacin and imipenem resistance and treatment in ICU settings were common features of non-biofilm-producing *A. baumannii* isolates [5]. Conversely, other studies have reported that microbial communities in an ICU setting are efficient biofilm producers [8,10].

Furthermore, our study reported that device-related isolates tended to be associated with significantly higher biofilm production, particularly in respiratory samples; however, this significant association was not observed among samples derived from other sources. These findings are concurrent with other observational and retrospective studies reporting that biofilm-producing strains isolated from the ventilators of patients receiving mechanical ventilation (endotracheal tube aspirates (ETA)), such as *A. baumannii* and *Pseudomonas aeruginosa,* displayed a significant positive correlation between the length of ventilation and biofilm production capacity [1,27,28]. A descriptive analytical study of 100 clinically suspected, ventilator-associated pneumonia cases curated and quantitatively assessed ETAs for the bacterial count and biofilm production, using the same three methods used herein [29]. The multi-variant analysis revealed that *Klebsiella pneumoniae* was the predominant bacterium isolated, followed by *A. baumannii,* with nearly 72% of isolates displaying strong or moderate biofilm formation [29]. Similarly, a multicenter cohort study assessed biofilm formation in 92 unrelated *A. baumannii* strains using a microtiter plate assay, indicating that biofilm-producing isolates were obtained from cases of catheter-related urinary infections, bloodstream infections, and shunt-related meningitis [5].

Herein, we performed ERIC-PCR to identify the molecular type of *A. baumannii* strains, of which 131 meropenem-resistant isolates are presented in Figure 4. Furthermore, fingerprint analysis identified the genotypic diversity of these isolates in different hospital locations. Few isolates were groupable at the 95% discrimination level (Table 3); however, these isolates were chronologically unrelated, making their direct spread less likely. Some genetically identical strains exhibited variable susceptibility patterns, particularly towards aminoglycosides (amikacin and gentamicin), with more recent strains (306, 277 and 316) being susceptible. This finding is potentially associated with variability in the selective pressure imposed by aminoglycoside use; thus, the elevated fitness cost resulting from resistance genes may lead to the loss of the mobile element harboring the resistance determinant or “switch off” their expression in the absence of selective pressure [30]. On examining a smaller subset of *Acinetobacter* isolates (*n* = 85), Al Sultan et al. reported a cluster of eight isolates with a variable resistance profile among similar clones [31]. Furthermore, previous local studies have reported carbapenem-resistant *A. baumannii* isolates (CRAB) belonging to diverse, ungroupable clones [32,33]. Most isolates in this cohort were MDR organisms; therefore, more studies, including fully susceptible isolates, are needed to compare the genetic determinants involved in biofilm formation in different *A. baumannii* strains. Further studies are required to determine the correlation between *A. baumannii* adherence, biofilm formation, and its tendency to cause outbreaks and life-threatening, invasive infections, and to analyze quantitative differences between biofilm formation in clinical isolates and their association with strain epidemicity and infection severity. Understanding the triggers of biofilm formation would help limit and contain biofilm-associated infections and facilitate biofilm-specific therapeutic measures. Qualitative and quantitative analysis of biofilm-related genes would further the current understanding of the molecular basis of biofilm development and may influence the treatment of biofilm-associated infections.

Together, the present results indicate the importance of adhering to the infection control measures and implementing effective antimicrobial stewardship programs to limit the dissemination of biofilm-related infections of MDR *A. baumannii* clones, particularly in critical hospital units [34].

## 4. Material and Methods

### 4.1. Strains Characterization

In total, 207 non-replicate *A. baumannii* isolates were prospectively and serially obtained from the clinical specimens and indwelling medical devices, including central lines, peripheral venous cannula, tracheal tubes, peritoneal dialysis catheters, urinary catheters, and cerebrospinal shunt, collected from patients of all age groups, admitted to a University Hospital between January 2016 and December 2018. The clinical samples were inoculated onto MacConkey agar and blood agar plates (SPML, KSA) and incubated overnight at 35 °C. Suspected *A. baumannii* isolates, based on their colony morphology and oxidase-negative, catalase-positive reactions, were further analyzed to confirm their identity.

### 4.2. Identification of Phenotypic Species and Antibiotic Susceptibility Testing (AST)

*A. baumannii* isolates were identified in a Diagnostic Microbiology laboratory using a VITEK MS system (bioMérieux, Craponne, France) based on matrix-assisted laser desorption/ionization time-of-flight (MALDI-TOF) technology. AST was performed using a VITEK 2 system (bioMérieux). E-tests (AB Biodisk, Solna, Sweden) were used to determine the minimum inhibitory concentrations (MICs) for carbapenems in accordance with the Clinical Laboratory Standards Institute (CLSI 2018) guidelines, while the MIC for tigecycline was estimated in accordance with US FDA breakpoints. The following control strains were included in each AST run: *Klebsiella pneumoniae* (ATCC 700603), *Escherichia coli* (ATCC 25922), and *Pseudomonas aeruginosa* (ATCC 27853). All isolates displaying an MDR profile, as defined by non-susceptibility to at least one agent in ≥3 antimicrobial categories, were further assessed [11]. Glycerol stocks of all bacterial isolates were stored at −80 °C prior to biofilm production analysis and molecular genotyping.

### 4.3. Detection of Biofilm Formation

#### 4.3.1. Tissue Culture Plate (TCP) Method

Tissue culture plate method was used as a reference method to screen MDR *A. baumannii* isolates [35]. Fresh bacterial cultures were inoculated into 10 mL of tryptic soy broth (TSB) supplemented with 1% glucose and incubated at 37 °C. Following overnight incubation, the cultures were diluted 1:100 using a fresh medium, and 0.2 mL aliquots were transferred into flat-bottomed microtiter plates and incubated for 24 h at 37 °C. The cells were then washed four times with 0.2 mL of PBS (pH 7.2) to remove the planktonic cells, and the bacteria forming biofilms at the bottom of the wells were subsequently fixed and stained with 2% sodium acetate and 0.1% crystal violet, respectively. After washing the wells with deionized water to remove the excess stain, the optical density of the solution was measured at 595 nm using an ELISA reader (Thermo Fisher Scientific Inc., Waltham, MA, USA). Independent experiments were performed in triplicate, and the results interpreted in accordance with Ansari et al. [36].

#### 4.3.2. Congo Red Agar (CRA) Method

*A. baumannii* biofilm formation was assessed in vitro using the CRA method described by Freeman et al. [37]. Briefly, freshly grown cultures were plated onto Brain Heart Infusion (BHI) Agar supplemented with Congo Red (0.8 g/L) and 5% sucrose, followed by aerobic incubation for 24–48 h at 37 °C. CRA-positive strains appeared as black colonies with a dry, crystalline consistency, while CRA-negative producers displayed darkening at the center of the colonies. Colony darkening without a dry crystalline morphology was interpreted as an indeterminate result, as previously described [36,37].

#### 4.3.3. Tube Method (TM)

The biofilm formation potential of clinical *A. baumannii* isolates was qualitatively assessed using the method of Christensen et al. [36,38]. Fresh bacterial growth was sub-cultured in 10 mL of tryptic soy broth (TSB) supplemented with 1% glucose in 25 mL tubes for 24 h at 37 °C. Following incubation, the tubes were washed with 1× phosphate-buffered saline (PBS; pH 7.3), dried and tested for evidence of biofilm formation, as indicated by the appearance of visible film lining the wall and bottom of the tube. Based on the intensity of the color formed, biofilm formation was scored as negative/weak (-/+), moderately positive (++), and strongly positive (+++) [36].

### 4.4. Genomic DNA Extraction, Enterobacterial Repetitive Intergenic Consensus (ERIC)-PCR Fingerprinting, and Cluster Analysis

Analysis of the clonal relatedness of pathogens could further the current understanding of molecular epidemiology and affect the infection control measures by monitoring the spread of strains and infection outbreaks. To extract genomic DNA, a single bacterial colony emulsified in 0.250 mL of molecular Biology grade water was incubated at 95 °C in a heating block for 15 min and then centrifuged at 13,000 rpm for 10 min. ERIC-PCR was carried out with 2 µL of the supernatant as the DNA template and ERIC1 (5′-TGT AAG CTC CTG GGG ATT CAC-3′) and ERIC2 (5′-AAG TAA GTG ACT GGG GTG AGC G-3′) primers, as described by Versalovic et al. [39], in a total volume of 25 µL. ERIC-PCR cycling conditions included initial denaturation at 94 °C for 5 min, 35 cycles at 95 °C for 1 min, 52 °C for 1 min, and 72 °C for 5 min, and a final extension at 72 °C for 10 min. Generated PCR products were loaded on 1.5% agarose gels stained with ethidium bromide and subject to electrophoresis to separate the bands. Cluster analysis was performed through pairwise comparisons of ERIC profiles using fingerprinting cluster analysis in the BioNumerics software package Version 7.6.3 (Applied Maths, Belgium). The generated dendrograms from the ERIC-PCR profiles, using Pearson’s correlation coefficient as a similarity measure and the unweighted pair group method (UPGMA) as a clustering algorithm with 1% optimization and 1% position tolerance, were used to identify isolates with a similarity exceeding 95% as clonally related.

### 4.5. Statistical Analyses

Statistical analyses were performed using Graphpad Prism Version 6.0 for Mac. Two-tailed *p*-values of <0.05 were considered statistically significant. Continuous variables were expressed in median and range values, whereas categorical variables were described as frequencies and percentages.

Fisher’s exact test was used to assess the relationship between categorical variables, namely, the association between the degree of biofilm formation and presence of indwelling medical devices at the site of infection, colonization versus infection, and the level of antimicrobial resistance, expressed as the number of drugs to which the pathogen is resistant.

### 4.6. Ethical Considerations

This study was a part of a project approved by the ethical committee of the Institutional Review Board at Imam Abdulrahman Bin Faisal University (IRB-2020-03-163). The material presented is original, unpublished, and has not been submitted elsewhere.

## 5. Conclusions

This study genotyped and analyzed the antibiotic resistance phenotype of various MDR *A. baumannii* isolates and used three different techniques to qualitatively estimate their biofilm formation potential. Although no significant association was observed between antibiotic resistance and adherence potential of isolates, ventilator-associated strains were potent biofilm producers. Additionally, strains derived from true infection cases appeared to have higher biofilm-producing potential than those collected from colonization sites. The frequent use of invasive medical devices, coupled with the extensive use of antimicrobial agents, could strongly drive selection for highly virulent and resistant isolates. Unfortunately, this cycle is hard to avoid and represents a significant risk of infection control. Large scale clinical studies on properties of biofilm-producing isolates may facilitate attempts to combat drug-resistant organisms.

## Figures and Tables

**Table 1:** Epidemiological data analysis.

	Number	%
Gender		
Male	128	61.8
Female	79	38.2
Age (years)		
1–14	7	3.4
15–44	54	26.1
45–74	94	45.4
≥75	52	25.1
Source *		
Respiratory tract	97	46.9
Skin and soft tissues	66	31.9
Blood	16	7.7
Others	28	13.5

* In this study, there were 96 device-related infections (8 central lines, 25 peripheral venous cannulas, 56 tracheal tubes, 4 urinary catheters, and 3 cerebrospinal shunts), while 8 strains originated from a device tip but were not subsequently isolated from the patient’s samples.

**Table 2:** Comparative analysis of tube method and Congo red agar method relative to the standard tissue culture plate method.

	Sensitivity (%)	Specificity (%)	PPV (%)	NPV (%)	Accuracy (%)
TM	52.73 (44.82–60.54)	72.22 (46.52–90.31)	96.21 (92.23–98.19)	10.31 (7.64–13.77)	54.09 (46.58–61.47)
CRA	6.51 (3.29–11.35)	92.86 (66.13–99.82)	92.37 (62.73–98.86)	6.96 (6.04–8.00)	12.55 (8.12–18.24)

Abbreviations: TM, tube method; CRA, Congo red agar; PPV, positive predictive value; NPV, negative predictive value.

**Table 3:** Clinical characteristics of the related *A. baumannii* clones.

Clone	Isolate	Date	Age	Sex	Location	Specimen	MRP	CAZ	TAZ	FEP	IMIP	CIP	LEVO	GENT	AMIK	TGC	TRIMETH	Biofilm
A1	128	May-18	30	F	Surgical Unit	Urine	R	R	R	R	R	R	R	R	R	S	R	strong
	306	Oct-18	63	M	medical ICU	Sputum	R	R	R	R	R	R	R	S	S	S	R	strong
A2	132	May-18	83	F	ER Unit	Rectal	R	R	R	R	R	R	R	R	R	S	R	intermediate
	304	Oct-18	19	F	Medical Unit	Throat	R	R	R	R	R	R	R	R	R	S	S	intermediate
A3	65	Feb-17	87	F	Medical ICU	Transtracheal	R	R	R	R	R	R	R	R	R	S	R	strong
	277	Aug-18	60	M	Medical Unit	Sacral	R	R	R	R	R	R	R	S	S	S	R	strong
A4	262	Jul-18	79	F	Medical ICU	Rectal	R	R	R	R	R	R	R	R	R	S	R	strong
	316	Nov-18	71	F	Medical ICU	Blood	R	R	R	R	R	R	R	S	S	S	S	strong

MRP: meropenem, CAZ: ceftazidime, TAZ: piperacillin/tazobactam, FEB: cefepime, IMP: imipenem, CIP: ciprofloxacin, LEVO: levofloxacin, GENT: gentamicin, AMIK: amikacin, TGC: tigecycline, and TRIMETH: trimethoprim.
